# Hybrid Resiliency-Stressor Conceptual Framework for Informing Decision Support Tools and Addressing Environmental Injustice and Health Inequities

**DOI:** 10.3390/ijerph16081466

**Published:** 2019-04-25

**Authors:** Kristen Burwell-Naney, Sacoby M. Wilson, Siobhan T. Whitlock, Robin Puett

**Affiliations:** 1 Center for Outreach in Alzheimer’s, Aging and Community Health, North Carolina A&T State University, 2105 Yanceyville Street, Greensboro, NC 27405, USA; 2 Maryland Institute for Applied Environmental Health, School of Public Heath, University of Maryland, 255 Valley Drive, College Park, MD 20742, USA; swilson2@umd.edu (S.M.W.); rpuett@umd.edu (R.P.); 3 Office of Environmental Justice and Sustainability, U.S. Environmental Protection Agency, 61 Forsyth Street SW, Atlanta, GA 30303, USA; whitlock.siobhan@epa.gov

**Keywords:** conceptual framework, resilience buffers, socio-environmental stressors, health inequities, screening tools, community-engaged research, cumulative risk assessment, allostatic load, salutogens, pathogens, environmental justice

## Abstract

While structural factors may drive health inequities, certain health-promoting attributes of one’s “place” known as salutogens may further moderate the cumulative impacts of exposures to socio-environmental stressors that behave as pathogens. Understanding the synergistic relationship between socio-environmental stressors and resilience factors is a critical component in reducing health inequities; however, the catalyst for this concept relies on community-engaged research approaches to ultimately strengthen resiliency and promote health. Furthermore, this concept has not been fully integrated into environmental justice and cumulative risk assessment screening tools designed to identify geospatial variability in environmental factors that may be associated with health inequities. As a result, we propose a hybrid resiliency-stressor conceptual framework to inform the development of environmental justice and cumulative risk assessment screening tools that can detect environmental inequities and opportunities for resilience in vulnerable populations. We explore the relationship between actual exposures to socio-environmental stressors, perceptions of stressors, and one’s physiological and psychological stress response to environmental stimuli, which collectively may perpetuate health inequities by increasing allostatic load and initiating disease onset. This comprehensive framework expands the scope of existing screening tools to inform action-based solutions that rely on community-engaged research efforts to increase resiliency and promote positive health outcomes.

## 1. Introduction

The environment is a multifaceted system as it relates to health, encompassing not only physical, chemical, and biological factors that are external to a person, but social and cultural aspects as well [1]. Salutogenic and pathogenic factors representing “the creation or origins of health” and “creation or origins of disease” respectively may further characterize the environment [2,3]. Specifically, salutogens are defined as components of an individual’s social, economic, physical, and emotional environment that may foster health and wellbeing while pathogens are attributes of one’s environment that may undermine health [2,3]. The interaction of these factors in the environment has significant implications for environmental justice (EJ) and cumulative risk assessment (CRA) screening tools, since salutogens (i.e., access to healthcare, green space, healthy food outlets) may have the ability to buffer cumulative impacts of multiple environmental exposures [4] and modify an individual’s actual risk or vulnerability profile. To effectively reduce risk and become resilient, individuals must have access to available assets (i.e., salutogens) in their communities and an ability to utilize them to function during and/or after a disturbance [5].

Socio-environmental stressors do not work independently and are often additive and/or multiplicative in nature; thereby creating cumulative impacts that may lead to adverse health outcomes over the life course. Exposures to environmental stressors modify aspects of gene expression in the epigenome that may cause individuals to deviate from a life course trajectory of optimal health [6]. Differentially burdened populations of color and economically marginalized groups are particularly vulnerable to epigenetic modifications since they often experience the greatest exposure to environmental hazards in their neighborhoods [7,8,9,10,11] and may lack health-promoting infrastructure or resiliency buffers that are necessary to offset negative exposures. Without these safeguards in place, communities of color and low-income groups overburdened by EJ issues (i.e., heavily trafficked roadways, Toxic Release Inventory [TRI] facilities, crime) may be more likely to have higher mortality rates of stroke, hypertension, cancer, diabetes, and heart disease when compared to their White and more affluent counterparts [12,13].

While many screening tools have evolved to include environmental stressors, they have not traditionally accounted for resilience factors that may modify the impact of exposure to chemical and non-chemical stressors. As a result, these screening tools may inaccurately depict risk and a community’s ability to overcome adverse exposures. This work proposes a hybrid resiliency-stressor conceptual framework that may be used to inform the development of EJ and CRA screening tools that can be used to identify opportunities for resilience within vulnerable communities. We explore various aspects of CRA and resilience, their proclivity to be addressed in silos, and the limitations of neglecting their synergistic relationship when designing EJ and CRA screening tools. We also explain the conceptual framework and variables that represent each domain. By considering the unique combination of variables described in our hybrid resiliency-stressor conceptual framework, we aim to broaden the discussion on how to better address environmental health inequities and cultivate resilient communities. In addition, our framework demonstrates how community-engaged research approaches and exposures to salutogens are the impetus to promoting community resilience and health equity.

### 1.1. Cumulative Risk Assessment Background

Improving our understanding of ways to eliminate environmental injustice and reduce health inequities requires the deliberate consideration of combined risks of chemical and non-chemical stressors on various populations over time. Previously, a single-chemical risk assessment approach was used to identify threats to human health through hazard identification, dose-response-assessment, exposure assessment, and risk characterization [14]. When conducting a single-chemical risk assessment, a specific chemical was often examined to determine its unique exposure pathways, media, or endpoints. The main limitation of this assessment methodology was its inability to evaluate vulnerability and multidimensional problems inclusive of chemical, biological, physical, and social stressors that are often modified when operating collectively [15]. This is particularly concerning in communities impacted by EJ issues since they are more likely to experience excess exposures to multiple socio-environmental stressors. Conventional risk assessment approaches no longer had the capacity to address the challenges of a complex and changing environment.

The US Environmental Protection Agency (EPA) responded to the constraints of conventional risk assessment methods by developing a *Framework for Cumulative Risk Assessment* to guide the process of understanding combined risks to human health and the environment from multiple agents or stressors. The essential components of CRAs include: (1) an ability to account for multiple stressors, (2) consideration of interactions between various stressors, and (3) a focus on how a multitude of stressors may impact populations versus individuals [16,17]. Other factors that may be incorporated into CRAs are different environmental media, as well as durations, pathways, and/or routes of exposure that may have a bearing on risk. Multiple effects, risks quantified from multiple chemical and non-chemical stressors, and concepts of vulnerability may also be included in CRAs [18]. Another important characteristic of CRAs are their use of stakeholder involvement or community engagement during the planning, scoping, and problem formulation phases [19].

### 1.2. Challenges of Operating in Cumulative Risk Assessment Silos

Though CRA has evolved from conventional risk assessment methods, there are still several challenges inherent in current methods that demonstrate the need to approach CRAs and respective tools from a systems science lens instead of operating in silos. Some of the challenges of CRAs involve characterizing multiple stressors with varying toxicities and pathways that lack a common endpoint [20] and being able to incorporate non-occupational and occupational exposures due to the variability in workplace stressors, interaction mechanisms, and duration of potential exposures [21]. Other challenges may be more systematic, such as not having a standardized metrics in place to measure exposures to non-chemical stressors across populations or not having data with the same units and time [21]. In addition, there may be limitations in quantifying the interactions between chemical and non-chemical stressors and determining the dose-response relationship for disease outcomes [22]. CRAs also fail to incorporate the impact of salutogenic factors that may act on a system, genetic susceptibilities, and often the perceptions of environmental factors that may negatively or positively influence health.

Despite these limitations, there are certain “best practices” that support dismantling CRA silos by using a systems science approach. A systems science approach to CRA would involve examining the interaction of various environmental factors (i.e., salutogenic and pathogenic) at different levels (i.e., individual, family, and community) using expertise from a multidisciplinary, multi-racial/ethnic, and multi-level SES group of stakeholders. Stakeholder engagement is an essential component of any CRA since community members provide a level of local expertise that may not be found in the literature [23]. Several studies have demonstrated how community-engaged research (i.e., community-based participatory research [CBPR], citizen science, community-owned and managed research [COMR]) can be used as a mechanism to address environmental injustice and health inequities in vulnerable populations [24,25,26,27,28,29,30,31].

Another study suggests developing a conceptual model during the problem formulation phase of a CRA to identify relationships between various stressors and assets that may impact risk [23]. While developing a conceptual model is not necessary for problem formulation, it can serve as a blueprint to inform various aspects of CRAs pertaining to the stakeholders and indicators that should be used when developing EJ screening tools to address health inequities. Current frameworks have tried to address many of the deficient characteristics of CRAs by considering the joint contribution of socio-environmental factors that may affect health and wellbeing throughout the trajectory of the life course [32]. Nevertheless, we continue to build upon these conceptual frameworks to improve the screening tools that are used to identify inequities, plan public health interventions, and change policies.

The challenges of operating in CRA silos has been the catalyst to unveiling opportunities to improve CRA and EJ screening tools that represent a more complete system of individual, family, and community exposures. These opportunities are endless as we continue to develop new methodologies, gain a better understanding of gene-environment interactions, and recognize the importance of including a diverse group of stakeholders to inform the tools. Most screening tools have the ability to quantify the negative impacts of environmental stressors on vulnerable communities using indices, community assessment maps, or a combination of the two metrics [33,34,35]. However, these tools often fail to consider measures of resilience that may have the ability to counteract the cumulative impacts of multiple environmental stressors [4]. By excluding resiliency buffers or salutogens from CRA and EJ screening tools, a community’s true risk profile cannot be fully illustrated.

### 1.3. Resilience in the Context of Environmental Justice

Resilience is defined as the “the ability of a community to use its assets to strengthen public health and healthcare systems and to improve the community’s physical, behavioral, and social health to withstand, adapt to, and recover from adversity” [36]. While there are many definitions of resilience, some of the most noteworthy definitions contain five quintessential concepts that may influence a community’s ability to recover from a disaster: (1) attribute, (2) continuing, (3) adaptation, (4) trajectory, and (5) comparability [37]. Based on these concepts, resilience is an intrinsic attribute within a community that continues throughout the life of the community [37]. In communities that experience more chronic threats to resilience, these changes may be detected on a gradual scale compared to a more absolute change in resilience from a single disaster. Communities should also be able to adapt to adverse situations and have a positive trajectory, regardless of whether the adaptation occurs in response to or in anticipation of a prospective stressor. An acute shock may require more structural changes to adapt to a stressor while the cumulative impacts of a chronic stressor may further demand systemic changes to improve community resilience. Moreover, resilience should be defined in a way that allows communities to predict or compare their ability to positively adapt to a stressor [37].

Since communities are unique entities with varying needs, it is important to use an approach that addresses community resilience from a local perspective that may indirectly affect change on a higher level. Studies have shown that prioritizing and meeting the needs of a community’s most vulnerable populations may foster macro-level improvements in overall resilience [38,39]. This ideology is particularly important in communities impacted by EJ issues or injustices, since EJ occurs when “people can realize their highest potential without experiencing the “isms”. EJ is supported by decent paying and safe jobs, quality schools and recreation, decent housing and adequate health care, democratic decision-making and personal empowerment; and communities free of violence, drugs and poverty. These are communities where both cultural and biological diversity are respected and highly revered and where distributed justice prevails” [40]. The communities who often experience environmental injustice are comprised of predominately persons of color and economically disadvantaged residents who often bear the brunt of cumulative exposures to chemical (i.e., heavily trafficked roadways, Superfund sites, and brownfields) and non-chemical stressors (i.e., crime, poverty, and segregation). When vulnerable communities lack resiliency, they may be more likely to have higher mortality rates for stroke, hypertension, cancer, diabetes, and heart disease when compared to their White and more affluent counterparts [12,13].

Consequently, a dose-response relationship may exist between exposure to stressors and allostatic load that can promote a heightened level of vulnerability [41]. Allostatic load may be defined as the cumulative biological tax on the body resulting from physiological responses to daily stressors [42]. Vulnerability is a function of exposure to risk and resilience as demonstrated by the following equation,

(1) $$V_{i}=f\left(exposure_{risk},\;R_{i}\right)$$

where V_i_ is vulnerability, f represents function, exposure_risk_ is indicative of one’s risk of exposure, and R_i_ is resilience. As a result, any changes in resilience or one’s risk of exposure may directly influence vulnerability [43]. While vulnerability and resilience are different yet complimentary in nature, research suggests both concepts should be integrated into one assessment tool [44]. A recent study affirmed the relationship between resilience and vulnerability, and further demonstrated the significance of including both concepts in a framework that can be used when performing community assessments to obtain a more complete profile of human health risk [45].

### 1.4. Resiliency-Stressor Conceptual Framework Overview

The resiliency-stressor conceptual framework was developed by performing a comprehensive literature review of factors that may influence resilience, promote stress, and perpetuate health disparities. The nomenclature of the conceptual framework is based on the synergistic relationship that exists between resilience buffers and stressors; therefore, both types of variables are represented to better understand human health risks. While this conceptual framework presents a more American health perspective of resilience and stressors, the same concepts may be applied to other parts of the world by altering some of the variables to reflect salutogens and pathogens in other countries.

**Figure 1:**
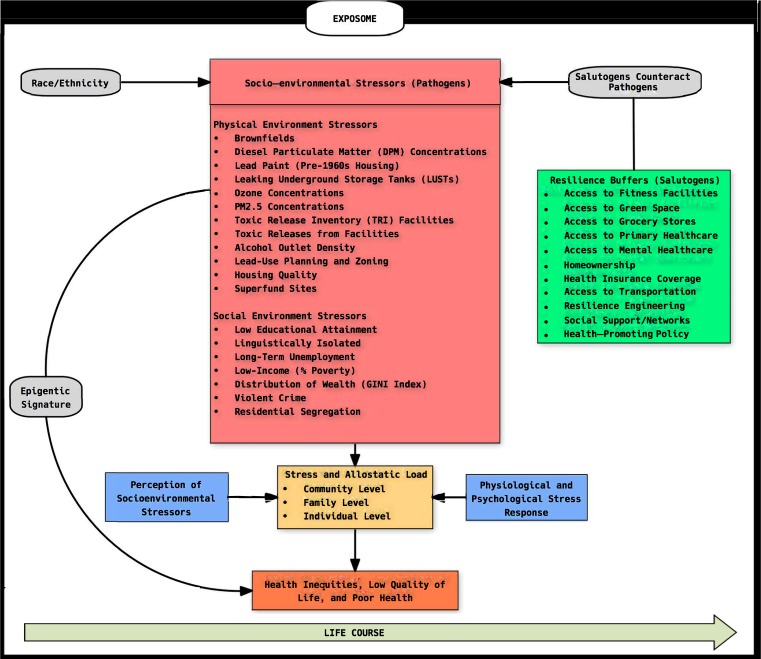
Resiliency-Stressor Conceptual Framework.

In our conceptual framework (Figure 1), we explore the relationship between actual exposures to socio-environmental stressors, perceptions of stressors, and one’s physiological and psychological stress response to environmental stimuli. These factors work collectively as a “three-legged stool” to perpetuate health inequities by increasing allostatic load and initiating disease onset [46]. We present several physical and social stressors as part of the socio-environmental stressors domain that may be pathogenic, as well as salutogens within the resiliency buffers domain that have the ability to counteract the negative impacts of the socio-environmental stressors and promote positive health outcomes [4]. Race/ethnicity is depicted as the culprit in driving or exacerbating the negative effects of socio-environmental stressors since studies have demonstrated how inequities in proximity to environmental hazards by race/ethnicity persist after controlling for other sociodemographic characteristics (i.e., income, education) [47]. We also provide evidence of how exposures to socio-environmental stressors may alter one’s epigenetic signature; hence, making them more susceptible to adverse health outcomes [48]. We further explain the components of the conceptual framework in subsequent sections.

## 2. Resiliency-Stressor Conceptual Framework Components

### 2.1. Exposome

An exposome layer encapsulates our resiliency-stressor conceptual framework (Figure 1), which is a concept first defined in 2005 as the totality of life-course environmental exposures from conception onwards [49]. The exposome concept considers the complexity of multiple environmental exposures and responses to chemical stressors, non-chemical stressors, microbiome metabolites, and infectious agents at varying levels and scales that may pose threats to human health [50,51]. Specifically, the exposome represents the cumulative environmental exposures (i.e., stress, diet, and air pollution) of an individual over their life-course that may have an impact on health. Since the exposome encapsulates gene-environment interactions over time, it has been documented as a major component in advancing CRAs [52]. In our conceptual framework, we include different variables that represent each of the three domains of non-genetic exposure characteristic of an exposome: (1) internal (i.e., stress), (2) specific external (i.e., chemical agents and environmental pollutants), and (3) general external (i.e., poverty, education) [53]. There are several approaches that can be used to measure aspects of the exposome like biomarkers, sensor technologies, or imaging, but for the purposes of our conceptual framework, we are using the term in a theoretical context to demonstrate the scale of exposures over the life-course. Within the exposome, we also demonstrate how exposures to environmental stressors, perceptions of environmental stressors, and resilience factors may work collectively to impact allostatic load (i.e., stress) at varying levels (i.e., individual, family, and community).

### 2.2. Stress and Allostatic Load

Allostatic load refers to the cumulative biological tax on the human body that may result from chronic exposures to daily stressors [42]. Consequently, a dose-response relationship may exist between exposures to environmental stressors and allostatic load that can promote vulnerability or risk [41]. This conceptual framework considers the relationship between cumulative environmental exposures, allostatic load, and disease onset, which may be influenced by an individual’s valuation of exposure and their accompanying physiological and psychological response to a stressor [46]. Allostatic load has been associated with damaging health behaviors (i.e., alcohol and tobacco abuse), cardiovascular disease, and mortality, which may further proliferate health inequities among vulnerable populations [54].

Social and environmental stressors play a critical role in perpetuating health inequities [13,55,56] due to their contribution to chronic individual stress and allostatic load [42,57,58]. In our conceptual framework, a stressor is considered as any perceived or actual threat to an individual that has the capacity to disrupt homeostasis and cause stress [59]. Most people experience acute stress in response to a stressful event over the life course; however, chronic stress may be more problematic since it can increase one’s risk for several health problems like heart disease [60], neurologic and psychiatric diseases [61], Parkinson’s disease [62], Alzheimer’s disease [63], multiple sclerosis [64], eating disorders [65], addictions [66], post-traumatic stress disorder (PTSD) [67], and sleeping complications [68].

### 2.3. Actual Socio-Environmental Stressors

Chronic exposures to socio-environmental stressors that are social and physical in nature may contribute to increases in allostatic load and promote inequities in health. For example, populations with less educational attainment may have a higher morbidity or mortality rate for stroke, high cholesterol, ulcers, asthma, hypertension, diabetes, and high cholesterol [69]. They may also be more likely to participate in risky behaviors (i.e., smoking, drinking) [70] and avoid healthy behaviors related to diet and exercise [71]. Similarly, linguistic isolation may be classified as a social stressor since populations speaking limited English may lack information on symptoms or services available for disease prevention or maintenance and consequently delay medical care [72]. Linguistically isolated populations may also be exposed to racial discrimination due to language barriers, which is a phenomenon that has been associated with low socioeconomic (SES), poor quality of life, and stress [73].

Regarding unemployment status, a few studies have shown unemployed individuals have worse psychological and physical health than those who are currently employed [74,75]. Moreover, unemployed individuals are more likely to delay health care services due to the financial burden and less likely to have access to health care that may be required to maintain wellbeing [75]. Unemployment is also associated with an increased risk of poverty [76], and economically disadvantaged populations often have higher mortality rates for almost all major causes of death (e.g., infectious, nutritional, cardiovascular, metabolic diseases, cancers, and injuries) [77,78]. In addition, they may have increased susceptibility to disease due to the adverse effects of chronic stress on the body’s immune system [79].

Income inequality models elucidate the chronic stress response of poverty in the body and have been associated with low levels of trust and social cohesion [80]. Income inequality has a positive relationship with violent crime [81], and violent crime is further associated with psychological distress. Violent crime promotes psychological distress through indirect pathways involving the community’s perceptions of neighborhood disorder and actual violence experienced in the neighborhood [82]. This social stressor not only impacts mental health, but exposure to violent crime may also be associated with certain birth outcomes such as small for gestational age [83], low birth weight (LBW) [84], and preterm birth [85].

In addition, several studies have noted the relationship between environmental health inequities and residential segregation that may perpetuate health inequalities among populations of color [8,86,87,88,89,90]. Segregation has been associated with an increased risk of negative health outcomes, such as mortality [91], preterm birth [92], LBW [93], asthma [94], cardiovascular disease [95], cancer [86], and sexually transmitted infections [88]. This increased risk for adverse health conditions is likely attributable to the disproportionate distribution of environmental hazards in communities of color and low-income communities [9,12,13,56].

Like the aforementioned social stressors, physical environment stressors have the same capacity to increase one’s susceptibility to adverse health conditions. For example, exposures to toxicants from environmental hazards like brownfields have been strongly associated with ‘not good health’, limiting long-term illness, and premature all-cause mortality [96]. Leaking underground storage tanks (LUSTs) may release non-carcinogenic contaminants (i.e., toluene, ethylbenzene, xylene, petroleum) that increase an individual’s risk of developing kidney or liver damage, lung dysfunction, hearing loss, eye irritation, memory loss, difficulty breathing, and fatigue [97]. LUSTs may also release carcinogenic compounds (i.e., benzene) that lead to increased bleeding and decreased immune function [98]. Superfund sites contain an array of chemical contaminants that may lead to decreased immune function, impaired hearing, and increased risk for cardiometabolic conditions, birth defects, anemia, and urinary tract disorders [7,99,100,101,102]. TRI facilities and toxic releases have been linked with a higher risk of infant mortality [103], respiratory (i.e., asthma) and neurological diseases [104], and cancer [105].

Physical stressors, such as exposures to particulate matter ≤2.5 microns (PM_2.5_), have been associated with a higher risk of respiratory (i.e., asthma) [106] and cardiovascular conditions [107], LBW [108], pre-mature mortality [109], and hospital and emergency department visits related to cardiometabolic conditions [110]. Diesel PM may cause airway irritation [111], inflammation [112], and exacerbate asthma [113] like the other air toxics, but may also increase one’s risk for lung cancer [114] and neurological effects [115]. High traffic density, while not a specific air toxic, may increase an individual’s exposure to carbon monoxide (CO), carbon dioxide (CO_2_), volatile organic compounds (VOCs), nitrogen oxides (NO_x_), as well as PM emissions. Heavily trafficked roadway emissions have been linked to several adverse health outcomes, including increased risk of asthma exacerbation [116], cardiac and pulmonary mortality [117], additional hospital admissions, and other respiratory symptoms [118].

Poor housing quality is another physical stressor that is extremely important for health and wellbeing due to its ability to contribute to various adverse health conditions. Not only are physical conditions (i.e., ventilation, heat, cold, lighting) important, there are also chemical, biological, and social conditions to consider as factors that may impact health [87]. Specifically, substandard housing conditions have been associated with asthma, respiratory problems, injuries, mental health issues, and lead-poisoning [119]. Lead-poisoning may occur from exposures to lead-contaminated dust and lead paint found in older homes (i.e., pre-1960s), which are the primary source of lead exposure among children in the US [120]. Exposure to lead has also been associated with developmental neurotoxicity in children, reproductive dysfunction in adults, as well as toxicity to the kidneys, blood, and endocrine systems in both children and adults [121].

Land use planning and zoning practices have also played a role in escalating exposures to neighborhood stressors and minimizing the presence of health-promoting infrastructure in communities with EJ issues [122,123,124]. These aspects of the social environment or neighborhood contextual factors (i.e., crime, social cohesion, and collective efficacy) have been linked with hypertension [125], obesity [126], and risky health behaviors (i.e., smoking) [127]. Density of alcohol outlets is one neighborhood contextual factor that negatively impacts health, where persons with greater access to alcohol outlets were more likely to consume unhealthy quantities of alcohol and have a hospital visit related to anxiety, stress, or depression [128]. Another study indicated alcohol-related hospitalizations and mortality were significantly higher in communities with a higher density of alcohol outlets [129]. Other studies have shown a positive association between a higher density of alcohol outlets and higher rates of violence, crime, drunk driving accidents, and pedestrian injuries [130].

### 2.4. Perceptions of Stressors and Stress Response

Perceptions of exposures to environmental stressors are a salient aspect of our framework due to its relationship with the body’s physiological and psychological stress response. While some environmental exposures directly affect the stress response (i.e., exposure to air toxics) [131], other environmental stressors may induce a physiological stress response from the way they are perceived and processed [68]. For example, one study demonstrated how community perceptions of health risks related to environmental exposures to a petrochemical complex in Texas City, Texas (TX) were associated with interleukin-6 and viral reactivation biological markers of stress [132]. Other physiological responses significantly associated with perceived stress have included increases in cortisol, triglycerides, dehydroepiandrosterone sulphate (DHEAS), and fasting glucose [133].

The brain’s perception of an environmental stimulus as a stressor may be moderated by one’s memory or previous experiences, current physiological state, traits, and genotype [68]. Furthermore, perceptions of environmental stressors may differ by race/ethnicity and lead to a heightened stress response in populations of color. Specifically, differentially burdened populations of color may exhibit greater levels of environmental concern or higher perceptions of environmental risks than their White counterparts [134]. While this disparity is likely due to the fact that African-Americans are often disproportionately exposed to negative environmental stimuli, they may also experience an additional layer of stress related to historical and/or current exposures to racism and discrimination [135,136]. Greater perceptions of socio-environmental risk coupled with disproportionate exposures to environmental hazards make populations of color especially vulnerable to stress and adverse health outcomes.

### 2.5. Resilience Buffers

Our conceptual framework is further comprised of resilience buffers that may counteract physiological and psychological responses to the cumulative impacts of environmental stressors at various levels (i.e., individual, family, community). Resiliency generally refers to one’s ability to withstand, adapt to, or recover from adversity, which can occur at both an individual and/or community level. To effectively respond to stressors and become resilient, communities must have available assets that are accessible so they can function during and/or after a disturbance [5]. These assets may consist of health-promoting infrastructure (i.e., healthy food outlets, green space, health care facilities) that is necessary to counteract the cumulative impacts of multiple environmental stressors [3,13,42,137]. When vulnerable communities lack resiliency, they may be more likely to have higher mortality rates for stroke, hypertension, cancer, diabetes, and heart disease when compared to their White and more affluent counterparts [12,13].

Green space is one example of a resilience buffer since access to this ecosystem service has been associated with a reduced risk of cardiovascular disease [138,139,140], mental health outcomes [140], and type 2 diabetes mellitus [141], as well as increases in resiliency and wellbeing from social interactions [140]. Similar to green space, grocery stores may provide communities with access to healthy foods necessary to lower disease risk for cardiovascular disease [142], cancer [143], and hypertension [144]. Other resiliency buffers like homeownership may lead to a creation of wealth, which may promote higher rates of civic engagement and improvements in health due to one’s ability to afford quality healthcare [145]. Homeownership also fosters residential stability, which indirectly leads to increased academic performance among children and social capital in adults [134]. Moreover, homeownership may allow individuals to reside in communities with better schools, social conditions, and physical environments with less crime [146].

Studies have further demonstrated an association between increased primary care availability and better health outcomes [11,147], as well as the necessity of these services in counteracting the negative impacts that lower socioeconomic conditions may have on health [148]. Since mental health is so intricately linked with physical health, access to these services may have important implications in improving physical health and wellbeing [149]. Insurance status may also have a significant bearing on health and wellbeing, since insured populations often receive more preventive and screening services leading to improvements in health [150] and reduced mortality [151]. This may be particularly important for diseases like cancer (i.e., breast, prostate, or colorectal cancer), where uninsured patients are more likely to die prematurely than patients with insurance due to the delay in diagnosis [152].

Aside from equity access to health-promoting resources, there are still infrastructure considerations that may impact community resiliency. For example, access to transportation where individuals live and work may influence active commuting behaviors such as walking, cycling, or using public transit in combination with walking or cycling [153,154]. Increased access to these dynamic transportation networks has been associated with direct and indirect health benefits like reductions in traffic emissions, increases in physical and mental health, and improved access to health care services and healthy food [154,155]. Policies enforced at the local, state, or national government level are also a critical component of resilience when they reinforce appropriate practices such as zoning that reduces residential exposures to environmental hazards [156].

## 3. Challenges of Integrating Resilience into Cumulative Risk Assessment Screening

There are a few challenges that may arise when integrating resilience factors into CRA and EJ screening tools; however, we have been able to overcome many of those obstacles through the development and implementation of a Cumulative Stressors and Resiliency Index (CSRI). The details of this screening tool have been published in a previous study, but the CSRI is a community-informed index tool that uses various socio-environmental stressors and resilience buffers to rank human and environmental for South Carolina (SC) census tracts [157]. This CRA tool can account for resilience factors that may counteract the negative impacts of exposures to socio-environmental stressors. One issue to consider when designing this type of index is which variables should be classified as salutogens versus pathogens. Once this designation has been established for all variables, the directionality of the variable should be incorporated into the model to determine whether high or low values are indicative of more resilient communities. For example, salutogenic variables in the index are health-promoting factors like access to primary healthcare or access to grocery stores. Alternatively, these same factors can also be categorized as pathogens if there is low or no access.

The same challenges characteristic of CRAs are inherent in modified CRAs that may contain resilience factors; however, the complexity of interaction mechanisms between stressors and chemicals now include the relationship between salutogens and pathogens at multiple levels. The synergistic or other possible effects (i.e., moderating, mediating) are still not completely understood, nor are the precise exposure doses to resilience buffers that are necessary to counteract the negative impacts of environmental exposures. Nevertheless, several studies have shown how a community’s assets (i.e., healthy food outlets, green space, health care facilities) may offset many of these impacts [3,13,42,137] and provides support for including resilience factors in CRA screening tools. As more evidenced-based information becomes available to better quantify these relationships, we will continue to push the science forward to address the lingering gaps in integrating resilience factors into CRA screening tools.

## 4. Public Health Implications

This conceptual framework has major public health implications since it involves strengthening CRA screening tools so they incorporate socio-environmental stressors and resilience factors from our proposed framework to more accurately examine risk and address health inequities. Improving the variable inputs for CRA and EJ screening tools as well as community-engaged research efforts that inform those inputs will produce more meaningful outputs. Specifically, these changes will allow us to: (1) better identify opportunities for improving resilience (high environmental stress/low resiliency), (2) develop a blueprint of communities that are thriving to serve as a model to advance less resilient communities, (3) improve equity by linking low-resourced communities with neighboring assets, and (4) promote community resilience and sustainability. By using our framework to expand the scope of CRA and EJ screening tools, we will now be equipped to develop action-based solutions that are individualized to meet the unique needs of any community. Nevertheless, action-based solutions for reducing risk should focus more on improving community resilience through asset building since communities impacted by EJ issues are not always able to evade environmental stressors.

Formulating action-based solutions to truly improve community resilience requires a concerted effort from various stakeholders; therefore, operating in silos to address issues of resilience has become an obsolete practice. As previously mentioned, community-engaged research is the cornerstone of any resiliency work that is further enhanced by collaborations with multi-level stakeholders that span many disciplines (i.e., government, public health, health care, environmental health). This concept of using a multidisciplinary team has been effectively applied to both acute stressors like natural disasters (i.e., floods, hurricanes, earthquakes) and chronic stressors (i.e., disenfranchised and marginalized neighborhood environments) that may challenge community resilience. For example, the Los Angeles County Community Disaster Resilience (LACCDR) project uses a collaborative partnership between the Los Angeles County Department of Public Health, local community members, academics, and business stakeholders to address issues of community resilience related to disaster preparedness [158]. Other communities have prioritized community-engaged research in their implementation strategies for improving community resilience to different environmental stressors like Baton Rouge, Louisiana [159], southeast Texas [160], and Charleston, SC [157].

Addressing resiliency in high-risk areas is known to foster macro-level improvements in overall community resilience [35], which emphasizes the importance of having screening tools with resiliency factors that can identify areas of high-risk. Some communities have already actively begun to strengthen resilience in their most vulnerable neighborhoods using CRA tools to help inform the process. For example, the California Environmental Justice Alliance has started a Green Zone Initiative in California to transform communities differentially burdened by environmental justice issues into thriving human ecosystems. Their model involves three main components: (1) identify differentially burdened communities using cumulative risk assessment tools (i.e., CalEnviroScreen), (2) advance visionary Green Zone policy, and (3) demonstrate the model on-the-ground with seven differentially burdened communities that serve as anchor campaigns to support the Initiative. While the Green Zones Initiative identifies toxic hotspots (component 1) and prioritizes these areas for resources, green development, and regulatory attention (component 2) [161], having resilience buffers pre-populated in the CRA screening tool could have truncated the process and increased efficiency in the way vulnerable communities were prioritized.

As developing Green Zones becomes the standard for achieving community resilience through action-based solutions, it is important that screening tools continue to evolve at the pace of stakeholders’ needs. The framework we introduced exemplifies a comprehensive CRA model, that when applied through a community-engaged research lens, could change the way we identify and prioritize high-risk communities. Future research should focus on updating CRA and EJ screening tools and methodologies based the concepts presented in our framework to incorporate both socio-environmental stressors and resiliency buffers. Though we were able to develop the CSRI in a previous study using this framework, our index was a pilot version that merely demonstrated how principles from our framework could be used to address gaps in current screening tools.

We are in the process of developing other versions of the CSRI that will incorporate greater stakeholder input through community-engaged research practices. The new versions of the CSRI will not only allow community stakeholders to participate in the variable selection process, they will also have an opportunity to prioritize the indicators in a way that contributes to one layer of the weighting feature. The second layer of weighting will be based on the correlation between socio-environmental stressors and resilience factors. Lastly, we plan to explore the possibility of including an additional layer that captures physiological/psychological stress responses. These modifications will allow CRAs to operate in more of a precision public health capacity, and may ultimately improve the efficiency of projects like the Green Zone Initiative and other action-based solutions that can increase community resilience and reduce health inequities.

## 5. Conclusions

This work introduced a hybrid resiliency-stressor conceptual framework that may be used to modify existing EJ and CRA screening tools or inform the development of new tools to identify resilience opportunities within vulnerable communities. While the indicators within this framework are adjustable, including both cumulative pathogens and salutogens within the same model are critical to understanding a community’s true health and environmental risk. When these factors are considered collectively with perceptions of socio-environmental stressors and physiological and psychological stress responses, we gain a better understanding of how differences in allostatic load may contribute to health inequities over the life course. The information presented in this framework will ultimately allow us to design better EJ and CRA screening tools that strengthen our capacity to address health inequities and improve community resilience. 

## References

[1] World Health Organization What is the Environment in the Context of Health?. http://www.who.int/quantifying_ehimpacts/publications/preventingdisease2.pdf.

[2] Antonovsky A. (1987). Unraveling the Mystery of Health: How People Manage Stress and Stay Well.

[3] Wilson S. (2009). An ecologic framework to study and address environmental justice and community health issues. Environ. Justice.

[4] Davis R., Cook D., Cohen L. (2005). A community resilience approach to reducing ethnic and racial disparities in health. Am. J. Public Health.

[5] Longstaff P.H., Armstrong N.J., Perrin K., Parker W.M., Hidek M.A. (2010). Building resilient communities: A preliminary framework for assessment. Homel. Secur. Aff..

[6] Olden K., Freudenberg N., Dowd J., Shields A.E. (2011). Discovering how environmental exposures alter genes could lead to new treatments for chronic illnesses. Health Aff..

[7] Burwell-Naney K., Zhang H., Samantapudi A., Jiang C., Dalemarre L., Rice L., Williams E., Wilson S. (2013). Spatial disparity in the distribution of superfund sites in South Carolina: An ecological study. Environ. Health.

[8] Rice L.J., Jiang C., Wilson S.M., Burwell-Naney K., Samantapudi A., Zhang H. (2014). Use of segregation indices, Townsend Index, and air toxics data to assess lifetime cancer risk disparities in Metropolitan Charleston, South Carolina, USA. Int. J. Environ. Res. Public Health.

[9] Wilson S.M., Fraser-Rahim H., Williams E., Zhang H., Rice L., Svendsen E., Abara W. (2012). Assessment of the distribution of toxic release inventory facilities in Metropolitan Charleston: An environmental justice case study. Am. J. Public Health.

[10] Wilson S., Zhang H., Burwell K., Samantapudi A., Dalemarre L., Jiang C., Rice L., Williams E., Naney C. (2013). Leaking underground storage tanks and environmental injustice: Is there a hidden and unequal threat to public health in South Carolina?. Environ. Justice.

[11] Wilson S., Zhang H., Jiang C., Burwell K., Rehr R., Murray R., Dalemarre L., Naney C. (2014). Being overburdened and medically underserved: Assessment of this double disparity for populations in the state of Maryland. Environ. Health.

[12] Tufts University Global Development and Environment Institute Website Environmental Justice: Income, Race, and Health. http://www.ase.tufts.edu/gdae/education_materials/modules/Environmental_Justice.pdf.

[13] Morello-Frosch R., Zuk M., Jerrett M., Shamasunder B., Kyle A.D. (2011). Understanding the cumulative impacts of inequalities in environmental health: Implications for policy. Health Aff..

[14] U.S. Environmental Protection Agency Website Framework for Cumulative Risk Assessment. https://www.epa.gov/sites/production/files/2014-11/documents/frmwrk_cum_risk_assmnt.pdf.

[15] Rhodes E.L. (2003). Environmental Justice in America: A New Paradigm.

[16] Sexton K. (2012). Cumulative risk assessment: An overview of methodological approaches for evaluating combined health effects from exposure to multiple environmental stressors. Int. J. Environ. Res. Public Health.

[17] Sexton K., Linder S.H. (2010). The role of cumulative risk assessment in decisions about environmental justice. Int. J. Environ. Res. Public Health.

[18] National Environmental Justice Advisory Council U.S. Environmental Protection Agency Website. Ensuring Risk Reduction in Communities with Multiple Stressors: Environmental Justice and Cumulative Risks/Impacts. https://www.epa.gov/sites/production/files/2015-04/documents/ensuringriskreducationnejac.pdf.

[19] MacDonell M.M., Haroun L.A., Teuschler L.K., Rice G.E., Hertzberg R.C., Butler J.P., Chang Y.-S., Clark S.L., Johns A.P., Perry C.S. (2013). Cumulative risk assessment toolbox: Methods and approaches for the practitioner. J. Toxicol..

[20] Israel B.D. (1995). An environmental justice critique of risk assessment. NYU Environ. Law J..

[21] Williams P.R.D., Dotson G.S., Maier A. (2012). Cumulative risk assessment (CRA): Transforming the way we assess health risks. Environ. Sci. Technol..

[22] Lewis A.S., Sax S.N., Wason S.C., Campleman S.L. (2011). Non-chemical stressors and cumulative risk assessment: An overview of current initiatives and potential air pollutant interactions. Int. J. Environ. Res. Public Health.

[23] Barzyk T.M., Wilson S., Wilson A. (2015). Community, state, and federal approaches to cumulative risk assessment: Challenges and opportunities for integration. Int. J. Environ. Res. Public Health.

[24] Jordan R.C., Sorensen A.E., Biehler D., Wilson S.M., LaDeau S. (2019). Citizen science and civic ecology: Merging paths to stewardship. J. Environ. Stud. Sci..

[25] Sorensen A.E., Jordan R.C., LaDeau S.L., Biehler D., Wilson S.M., Pitas J.H., Leisnham P.T. (2019). Reflecting on efforts to design an inclusive citizen science project in West Baltimore. Citiz. Sci. Theory Pract..

[26] Commodore A., Wilson S., Muhammad O., Svendsen E., Pearce J. (2017). Community-based participatory research for the study of air pollution: A review of motivations, approaches, and outcomes. Environ. Monit. Assess..

[27] Brandt H.M., Haynes V.E., Rice L.J., Campbell D., Williams E., Wilson S.M., Glover S. (2017). Using photovoice as a tool for community engagement to assess the environment and explore environmental health disparities. J. Health Disparities Res. Pract..

[28] Wilson S., Aber A., Ravichandran V., Wright L., Muhammad O. (2017). Soil contamination in urban communities impacted by industrial pollution and goods movement activities. Environ. Justice.

[29] Burwell-Naney K., Wilson S.M., Tarver S.L., Svendsen E., Jiang C., Ogunsakin O.A., Zhang H., Campbell D., Fraser-Rahim H. (2017). Baseline air quality assessment of goods movement activities before the Port of Charleston expansion: A community–university collaborative. Environ. Justice.

[30] Wilson S., Campbell D., Dalemarre L., Fraser-Rahim H., Williams E. (2014). A critical review of an authentic and transformative community-university partnership. Int. J. Environ. Res. Public Health.

[31] Campbell R.L., Caldwell D., Hopkins B., Heaney C.D., Wing S., Wilson S.M., O’Shea S., Yeatts K. (2013). Integrating research and community organizing to address water and sanitation concerns in a community bordering a landfill. J. Environ. Health.

[32] Olvera Alvares H.A., Appleton A.A., Fuller C.H., Belcourt A., Kubzansky L.D. (2018). An integrated socio-environmental model of health and well-being: A conceptual framework exploring the joint contribution of environmental and social exposures to health and disease over the life span. Curr. Environ. Health Rep..

[33] Huang G., London J.K. (2012). Cumulative environmental vulnerability and environmental justice in California’s San Joaquin Valley. Int. J. Environ. Res. Public Health.

[34] OEHHA Update to the California Communities Environmental Health Screening Tool. https://oehha.ca.gov/media/downloads/calenviroscreen/report/ces3report.pdf.

[35] Su J.G., Morello-Frosch R., Jesdale B.M., Kyle A.D., Shamasunder B., Jerrett M. (2009). An index for assessing demographic inequalities in cumulative environmental hazards with application to Los Angeles, California. Environ. Sci. Technol..

[36] U.S. Department of Health and Human Services Community Resilience. http://www.phe.gov/Preparedness/planning/abc/Pages/community-resilience.aspx.

[37] Community & Regional Resilience Institute Definitions of Community Resilience: An Analysis. http://www.resilientus.org/wp-content/uploads/2013/08/definitions-of-community-resilience.pdf.

[38] Chandra A., Williams M., Plough A., Stayton A., Wells K.B., Horta M., Tang J. (2013). Getting actionable about community resilience: The Los Angeles County Community Disaster Resilience project. Am. J. Public Health.

[39] Wulff K., Donato D., Lurie N. (2015). What is health resilience and how can we build it?. Annu. Rev. Public Health.

[40] Bryant B. (1995). Environmental Justice: Issues, Policies and Solutions.

[41] Theall K.P., Drury S.S., Shirtcliff E.A. (2012). Cumulative neighborhood risk of psychosocial stress and allostatic load in adolescents. Am. J. Epidemiol..

[42] Sexton K., Linder S.H. (2011). Cumulative risk assessment for combined health effects from chemical and nonchemical stressors. Am. J. Public Health.

[43] Food and Agriculture Organization Resilience Index Measurement and Analysis Model. http://www.fao.org/3/a-i1402e.pdf.

[44] Miller F., Osbahr H., Boyd E., Thomalla F., Bharawani S., Ziervogel G., Walker B., Birkmann J., van der Leeuw S., Rockström J. (2010). Resilience and vulnerability: Complementary or conflicting concepts. Ecol. Soc..

[45] Bergstrand K., Mayer B., Brumback B., Zhang Y. (2015). Assessing the relationship between social vulnerability and community resilience to hazards. Soc. Indic. Res..

[46] Adler T. (2009). A complex relationship: Psychosocial stress, pollution, and health. Environ. Health Persp..

[47] Crowder K., Downey L. (2010). Inter-neighborhood migration, race, and environmental hazards: Modeling micro-level processes of environmental inequality. AJS.

[48] Perera F., Herbstman J. (2011). Prenatal environmental exposures, epigenetics, and disease. Reprod. Toxicol..

[49] Wild C.P. (2005). Complementing the genome with an “exposome”: The outstanding challenge of environmental exposure measurement in molecular epidemiology. Cancer Epidem. Biomar..

[50] Cui Y., Balshaw D.M., Kwok R.K., Thompson C.L., Collman G.W., Birnbaum L.S. (2016). The exposome: Embracing the complexity for discovery in environmental health. Environ. Health Persp..

[51] Juarez P., Matthews-Juarez P., Hood D., Im W., Levine R.S., Kilbourne B.J., Langston M.A., Al-Hamdan M.Z., Crosson W.L., Estes M.G. (2014). The public health exposome: A population-based, exposure science approach to health disparities research. Int. J. Environ. Res. Public Health.

[52] Smith M.T., de la Rosa R., Daniels S.I. (2015). Using exposomics to assess cumulative risks and promote health. Environ. Mol. Mutagen..

[53] Wild C.P. (2012). The exposome: From concept to utility. Int. J. Epidemiol..

[54] Beckie T.M. (2012). A systematic review of allostatic load, health, and health disparities. Biol. Res. Nurs..

[55] Clougherty J.E., Kubzansky L.D. (2009). A framework for examining social stress and susceptibility to air pollution in respiratory health. Environ. Health Perspect..

[56] Gee G.C., Payne-Sturges D.C. (2004). Environmental health disparities: A framework integrating psychosocial and environmental concepts. Environ. Health Perspect..

[57] Logan J.G., Barksdale D.J. (2008). Allostatis and allostatic load: Expanding the discourse on stress and cardiovascular disease. J. Clin. Nurs..

[58] Morello-Frosch R., Shenassa E.D. (2006). The environmental “riskscape” and social inequality: Implications for explaining maternal and child health disparities. Environ. Health Perspect..

[59] Schneiderman N., Ironson G., Siegel S.D. (2005). Stress and health: Psychological, behavioral, and biological determinants. Ann. Rev. Clin. Psychol..

[60] Torpy J.M., Lynm C., Glass R.M. (2007). Chronic stress and the heart. JAMA.

[61] Davis M.T., Holmes S.E., Pietrzak R.H., Esterlis I. (2017). Neurobiology of chronic stress-related psychiatric disorders. Chronic Stress.

[62] Austin K.W., Ameringer S.W., Cloud L.J. (2016). An integrated review of psychological stress in Parkinson’s disease: Biological mechanisms and symptom and health outcomes. Parkinsons Dis..

[63] Machado A., Herrera A.J., de Pablos R.M., Espinosa-Oliva A.M., Sarmiento M., Ayala A., Venero J.L., Santiago M., Villarán R.F., Delgado-Cortés M.J. (2014). Chronic stress as a risk factor for Alzheimer’s disease. Rev. Neurosci..

[64] Mohr D.C., Hart S.L., Julian L., Cox D., Pelletier D. (2004). Association between stressful life events and exacerbation in multiple sclerosis: A meta-analysis. BMJ.

[65] Yau Y.H.C., Potenza M.N. (2013). Stress and eating behaviors. Minerva Endocrinol..

[66] Sinha R. (2001). How does stress increase risk of drug abuse and relapse?. Psychopharmacology.

[67] McFarlane A.C. (2010). The long-term costs of traumatic stress: Intertwined physical and psychological consequences. World Psychiatry.

[68] Oken B.S., Chamine I., Wakeland W. (2015). A systems approach to stress, stressors and resilience in humans. Behav. Brain Res..

[69] Choi A.I., Weekley C.C., Chen S.-C., Li S., Tamura M.K., Norris K.C., Shlipak M.G. (2011). Association of educational attainment with chronic disease and mortality: The Kidney Early Evaluation Program (KEEP). Am. J. Kidney Dis..

[70] Pampel F.C., Krueger P.M., Denney J.T. (2010). Socioeconomic disparities in health behaviors. Annu. Rev. Sociol..

[71] Darmon N., Drewnowski A. (2008). Does social class predict quality?. Am. J. Clin. Nutr..

[72] Shi L., Tsai J., Higgins P.C., Lebrun L.A. (2009). Racial/ethnic and socioeconomic disparities in access to care and quality of care for US health center patients compared with non-health center patients. J. Ambul. Care Manag..

[73] Gee G.C., Ponce N. (2010). Associations between racial discrimination, limited English proficiency, and health-related quality of life among 6 Asian ethnic groups in California. Am. J. Public Health.

[74] McKee-Ryan F., Song Z., Wanberg C.R., Kinicki A.J. (2005). Psychological and physical well-being during unemployment: A meta-analytic study. J. Appl. Psychol..

[75] Pharr J.R., Moonie S., Bungum T.J. (2011). The impact of unemployment on mental and physical health, access to health care and health risk behaviors. ISRN.

[76] Saunders P. (2002). The Direct and Indirect Effects of Unemployment on Poverty and Inequality.

[77] Kawachi I., Kennedy B.P., Lochner K., Prothrow-Stith D. (1997). Social capital, income inequality, and mortality. Am. J. Public Health.

[78] Mansfield C., Novick L.F. (2012). Poverty and health: Focus on North Carolina. N. C. Med. J..

[79] Brunner E., Marmot M., Marmot M. (2005). Social organization, stress and health. Social Determinants of Health.

[80] Pickett K.E., Wilkinson R.G. (2015). Income inequality and health: A causal review. Soc. Sci. Med..

[81] Enamorado T., Lopez-Calva L.-F., Rodriguez-Castelan C., Winkler H. (2016). Income inequality and violent crime: Evidence from Mexico’s drug war. J. Dev. Econ..

[82] Curry A., Latkin C., Davey-Rothwell M. (2008). Pathways to depression: The impact of neighborhood violent crime on inner-city residents in Baltimore, Maryland, USA. Soc. Sci. Med..

[83] Masi C.M., Hawkley L.C., Piotrowski Z.H., Pickett K.E. (2007). Neighborhood economic disadvantage, violent crime, group density, and pregnancy outcomes in a diverse, population. Soc. Sci. Med..

[84] Messer L.C., Kaufman J.S., Dole N., Herring A., Laraia B.A. (2006). Violent crime exposure classification and adverse birth outcomes: A geographically-defined cohort study. Int. J. Health Geogr..

[85] Messer L.C., Kaufman J.S., Dole N., Herring A., Laraia B.A. (2006). Neighborhood crime, deprivation, and preterm birth. Ann. Epidemiol..

[86] Morello-Frosch R., Jesdale B.M. (2006). Separate and unequal: Residential segregation and estimated cancer risks associated with ambient air toxics in US Metropolitan areas. Environ. Health Perspect..

[87] Jacobs D.E. (2011). Environmental health disparities in housing. Am. J. Public Health.

[88] Kramer M.R., Hogue C.R. (2009). Is segregation bad for your health?. Epidemiol. Rev..

[89] Morello-Frosch R., Lopez R. (2006). The riskscape and the color line: Examining the role of segregation in environmental health disparities. Environ. Res..

[90] Landrine H., Corral I. (2009). Separate and unequal: Residential segregation and black health disparities. Ethn. Dis..

[91] Yang T.C., Matthews S.A. (2015). Death by segregation: Does the dimension of racial segregation matter?. PLoS ONE.

[92] Messer L.C., Laraia B.A., Mendola P. (2009). Segregation and preterm birth: The effects of neighborhood racial composition in North Carolina. Health Place.

[93] Debbink M.P., Bader M.D.M. (2011). Racial residential segregation and low birth weight in Michigan’s Metropolitan areas. Am. J. Public Health.

[94] Pearlman D.N., Zierler S., Meersman S., Kim H.K., Viner-Brown S.I., Caron C. (2006). Race disparities in childhood asthma: Does where you live matter?. J. Natl. Med. Assoc..

[95] Kershaw K.N., Albrecht S.S. (2015). Racial/ethnic residential segregation and cardiovascular disease risk. Curr. Cardiovasc. Risk Rep..

[96] Bambra C., Robertson S., Kasim A., Smith J., Cairns-Nagi J.M., Copeland A., Finlay N., Johnson K. (2014). Healthy land? An examination of the area-level association between brownfield land and morbidity and mortality in England. Environ. Plan. A.

[97] Wilson S.M., Fraser-Rahim H., Zhang H., Williams E.M., Samantapudi A.V., Ortiz K., Abara W., Sakati W. (2012). The spatial distribution of leaking underground storage tanks in Charleston, South Carolina: An environmental justice analysis. Environ. Justice.

[98] Agency for Toxic Substances and Disease Registry Toxicological Profile for Benzene. https://www.atsdr.cdc.gov/toxprofiles/tp3.pdf.

[99] Currie J., Greenstone M., Moretti E. (2011). Superfund cleanups and infant health. Am. Econ. Rev..

[100] Kouznetsova M., Huang X., Ma J., Lessner L., Carpenter D.O. (2007). Increased rate of hospitalization for diabetes and residential proximity of hazardous waste sites. Environ. Health Perspect..

[101] Lybarger J.A., Lee R., Vogt D.P., Perhac R.M., Spengler R.F., Brown D.R. (1998). Medical costs and lost productivity from health conditions at volatile organic compound-contaminated superfund sites. Environ. Res..

[102] Sergeev A.V., Carpenter D.O. (2010). Increased hospitalizations for ischemic stroke with comorbid diabetes and residential proximity to sources of organic pollutants: A 12-year population-based study. Neuroepidemiology.

[103] Currie J., Schmieder J.F. (2009). Fetal exposure to toxic releases and infant health. Am. Econ. Rev..

[104] Legot C., London B., Shandra J., Rosofsky A. (2011). Proximity to Industrial Releases of Toxins and Childhood Respiratory, Developmental, and Neurological Diseases: Environmental Ascription in East Baton Rouge Parish.

[105] Luo J., Hendryx M., Ducatman A. (2011). Association between six environmental chemicals and lung cancer incidence in the United States. J. Environ. Public Health.

[106] Xing Y.-F., Xu Y.-H., Shi M.-H., Lian Y.-X. (2016). The impact of PM2.5 on the human respiratory system. J. Thorac. Dis..

[107] Scheers H., Jacobs L., Casas L., Nemery B., Nawrot T.S. (2015). Long-term exposure to particulate matter air pollution is a risk factor for stroke: Meta-analytical evidence. Stroke.

[108] Ebisu K., Bell M.L. (2012). Airborne PM_2.5_ chemical components and low birth weight in the Northeastern and Mid-Atlantic regions of the United States. Environ. Health Perspect..

[109] Wang J., Xing J., Mathur R., Pleim J.E., Wang S., Hogrefe C., Gan C.M., Wong D.C., Hao J. (2017). Historical trends in PM_2.5_-related premature mortality during 1990–2010 across the Northern Hemisphere. Environ. Health Perspect..

[110] Kloog I., Coull B.A., Zanobetti A., Koutrakis P., Schwartz J.D. (2012). Acute and chronic effects of particles on hospital admissions in New-England. PLoS ONE.

[111] U.S. Environmental Protection Agency (2002). Health Assessment Document for Diesel Engine Exhaust.

[112] Xu Y., Barregard L., Nielsen J., Gudmundsson A., Wierzbicka A., Axmon A., Jönsson B.A., Kåredal M., Albin M. (2013). Effects of diesel exposure on lung function and inflammation biomarkers from airway and peripheral blood of healthy volunteers in a chamber study. Part. Fibre Toxicol..

[113] McEntee J.C., Ogneva-Himmelberger Y. (2008). Diesel particulate matter, lung cancer, and asthma incidences along major traffic corridors in MA, USA: A GIS analysis. Health Place.

[114] Silverman D.T. (2017). Diesel exhaust causes lung cancer—Now what?. Occup. Environ. Med..

[115] Kilbum K.H. (2000). Effects of diesel exhaust on neurobehavioral and pulmonary functions. Arch. Environ. Health.

[116] Brown M.S., Sarnat S.E., DeMuth K.A., Brown L.A.S., Whitlock D.R., Brown S.W., Tolbert P.E., Fitzpatrick A.M. (2012). Residential proximity to a major roadway is associated with features of asthma control in children. PLoS ONE.

[117] Brugge D., Durant J.L., Rioux C. (2007). Near-highway pollutants in motor vehicle exhaust: A review of epidemiologic evidence of cardiac and pulmonary health risks. Environ. Health.

[118] Cook A.G., deVos A.J.B.M., Pereira G., Jardine A., Weinstein P. (2011). Use of a total traffic count metric to investigate the impact of roadways on asthma severity: A case-control study. Environ. Health.

[119] Hood E. (2005). Dwelling disparities: How poor housing leads to poor health. Environ. Health Perspect..

[120] Agency for Toxic Substances & Disease Registry Lead toxicity: Where is lead found?. https://www.atsdr.cdc.gov/csem/csem.asp?csem=34&po=5.

[121] Sanborn M.D., Abelsohn A., Campbell M., Weir E. (2002). Identifying and managing adverse environmental health effects: 3. Lead exposure. Can. Med. Assoc. J..

[122] Maantay J. (2001). Zoning, equity, and public health. Am. J. Public Health.

[123] Wilson S., Hutson M., Mujahid M. (2008). How planning and zoning contribute to inequitable development, neighborhood health, and environmental injustice. Environ. Justice.

[124] Wilson S.M., Heaney C.D., Cooper J., Wilson O. (2008). Built environment issues in unserved and underserved African-American neighborhoods in North Carolina. Environ. Justice.

[125] Morenoff J.D., House J.S., Hansen B.B., Williams D.R., Kaplan G.A., Hunte H.E. (2007). Understanding social disparities in hypertension prevalence, awareness, treatment, and control: The role of neighborhood context. Soc. Sci. Med..

[126] Burdette H.L., Wadden T.A., Whitaker R.C. (2006). Neighborhood safety, collective efficacy, and obesity in women with young children. Obesity.

[127] Mmari K., Lantos H., Blum R., Brahmbhatt H., Sangowawa A., Yu C., Delany-Moretlwe S. (2014). A global study on the influence of neighborhood contextual factors on adolescent health. J. Adolesc. Health.

[128] Pereira G., Wood L., Foster S., Haggar F. (2013). Access to alcohol outlets, alcohol consumption and mental health. PLoS ONE.

[129] Richardon E.A., Hill S.E., Mitchell R., Pearce J., Shortt N.K. (2015). Is local alcohol outlet density related to alcohol-related morbidity and mortality in Scottish cities?. Health Place.

[130] Campbell C.A., Hahn R.A., Elder R., Brewer R., Chattopadhyay S., Fielding J., Naimi T.S., Toomey T., Lawrence B., Middleton J.C. (2009). The effectiveness of limiting alcohol density as a means of reducing excessive alcohol consumption and alcohol-related harms. Am. J. Prev. Med..

[131] Oeder S., Kanashova T., Sippula O., Sapcariu S.C., Streibel T., Arteaga-Salas J.M., Passig J., Dilger M., Paur H.R., Schlager C. (2015). Particulate matter from both heavy fuel oil and diesel fuel shipping emissions show strong biological effects on human lung cells at realistic and comparable in vitro exposure conditions. PLoS ONE.

[132] Peek M.K., Cutchin M.P., Freeman D., Stowe R.P., Goodwin J.S. (2009). Environmental hazard and stress: Evidence from the Texax City Stress and Health Study. J. Epidemiol. Commun. Health.

[133] Goldman N., Glei D.A., Seplaki C., Liu I.W., Weinstein M. (2005). Perceived stress and phsyiological dysregulation in older adults. Stress.

[134] Chakraborty J., Collins T.W., Grineski S.E., Maldonado A. (2017). Racial differences in perceptions of air pollution health risk: Does environmental exposure matter?. Int. J. Environ. Res. Public Health.

[135] Harrell J.P., Hall S., Taliaferro J. (2003). Physiological responses to racism and discrimination: An assessment of the evidence. Am. J. Public Health.

[136] Clark R., Anderson N.B., Clark V.R., Williams D.R. (1999). Racism as a stressor for African Americans. Am. Psychol..

[137] Hutson M.A., Wilson S. (2011). The role of community-based strategies in addressing metropolitan segrgation and racial health disparities. Community Dev. J..

[138] Richardson E.A., Pearce J., Mitchell R., Kingham S. (2013). Role of physical activity in the relationship between urban green space and health. Public Health.

[139] World Health Organization-Europe (2016). Urban Green Spaces and Health.

[140] Lee A.C.K., Jordan H.C., Horsley J. (2015). Value of urban green spaces in promoting healthy living and wellbeing: Prospects for planning. Risk Manag. Healthc. Policy.

[141] Astell-Bur T., Feng X., Kolt G.S. (2014). Green space is associated with walking and moderate-to-vigorous physical activity (MVPA) in middle-to-older-aged-adults: Findings from 203 883 Australians in the 45 and up study. Br. J. Sport Med..

[142] Liu S., Manson J.E., Lee I.-M., Cole S.R., Hennekens C.H., Willett W.C., Buring J.E. (2000). Fruit and vegetable intake and risk of cardiovascular disease: The women’s health study. Am. J. Clin. Nutr..

[143] Boeing H., Bechthold A., Bub A., Ellinger S., Haller D., Kroke A., Leschik-Bonnet E., Müller M.J., Oberritter H., Schulze M. (2012). Critical review: Vegetables and fruit in the prevention of chronic diseases. Eur. J. Nutr..

[144] Borgi L., Muraki I., Satija A., Willett W.C., Rimm E.B., Forman J.P. (2016). Fruit and vegetable consumption and the incidence of hypertension in three prospective cohort studies. Hypertension.

[145] Rohe W.M., Lindblad M. (2014). Reexamining the Social Benefits of Homeownership after the Housing Crisis.

[146] Ni J., Decker C. (2009). The impact of homeownership on criminal activity: Empirical evidence from United States’ county level data. Econ. Bus. J. Inq. Perspect..

[147] Chang C.-H., Stukel T.A., Flood A.B., Goodman D.C. (2011). Primary care physician workforce and medicare beneficiaries’ health outcomes. JAMA.

[148] Shi L. (2012). The impact of primary care: A focused review. Scientifica.

[149] World Health Organization Promoting Mental Health. http://www.who.int/mental_health/evidence/en/promoting_mhh.pdf.

[150] Sommers B.D., Gawande A.A., Baicker K. (2017). Health insurance coverage and health—What the recent evidence tells us. N. Engl. J. Med..

[151] Woolhandler S., Himmelstein D.U. (2017). The relationship of health insurance and mortality: Is lack of insurance deadly?. Ann. Intern. Med..

[152] Ozbay F., Johnson D.C., Dimoulas E., Morgan C.A., Charney D., Southwick S. (2007). Social support and resilience to stress: From neurobiology to clinical practice. Psychiatry.

[153] Dalton A.M., Jones A.P., Panter J.R., Ogilvie D. (2013). Neighbourhood, route and workplace-related environmental characteristics predict adults’ mode of travel to work. PLoS ONE.

[154] Victoria Transport Policy Institute Evaluating Public Transportation Health Benefits. http://www.apta.com/resources/reportsandpublications/Documents/APTA_Health_Benefits_Litman.pdf.

[155] Litman T. (2013). Transportation and public health. Annu. Rev. Public Health.

[156] The National Academies (2012). Disaster Resilience. A National Imperative.

[157] Burwell-Naney K., Wilson S.M., He X., Sapkota A., Puett R. (2018). Development of a cumulative stressors and resiliency index to ecamine environmental health risk: A South Carolina assessment. Environ. Justice.

[158] Bromley E., Eisenman D.P., Magana A., Williams M., Kim B., McCreary M., Chandra A., Wells K.B. (2017). How do communities use a participatory public health approach to build resilience? The Los Angeles County community disaster resilience project. Int. J. Environ. Res. Public Health.

[159] Keegan R., Grover L.T., Patron D., Sugarman O.K., Griffith K., Sonnier S., Springgate B.F., Jumonville L.C., Gardner S., Massey W. (2018). Case study of resilient Baton Rouge: Applying depression collaborative care and community planning to disaster recovery. Int. J. Environ. Res. Public Health.

[160] Kennedy M., Gonick S., Meischke H., Rios J., Errett N.A. (2019). Building back better: Local health department engagement and integration of health promotion and Hurricane Harvey recovery planning and implementation. Int. J. Environ. Res. Public Health.

[161] California Environmental Justice Alliance Green Zones. https://caleja.org/what-we-do/greenzones/.

